# Neuroserpin Inclusion Bodies in a FENIB Yeast Model

**DOI:** 10.3390/microorganisms9071498

**Published:** 2021-07-13

**Authors:** Valentina Vapore, Corrado Mazzaglia, Diego Sibilia, Mara Del Vecchio, Gernot Fruhmann, Marta Valenti, Elena Miranda, Teresa Rinaldi, Joris Winderickx, Cristina Mazzoni

**Affiliations:** 1 Department of Biology and Biotechnologies ‘Charles Darwin’, Sapienza University of Rome, 00185 Rome, Italy; valentina.vapore@uniroma1.it (V.V.); cm986@cam.ac.uk (C.M.); sibilon@gmail.com (D.S.); martva02@ucm.es (M.V.); mariaelena.mirandabanos@uniroma1.it (E.M.); teresa.rinaldi@uniroma1.it (T.R.); 2 Functional Biology, KU Leuven, 3000 Leuven, Belgium; mara.delvecchio@kuleuven.be (M.D.V.); gernot.fruhmann@gmail.com (G.F.); joris.winderickx@kuleuven.be (J.W.); 3 Pasteur Institute—Cenci Bolognetti Foundation, Sapienza University of Rome, 00185 Rome, Italy

**Keywords:** yeast, neurodegeneration, FENIB, protein aggregation

## Abstract

FENIB (familial encephalopathy with neuroserpin inclusion bodies) is a human monogenic disease caused by point mutations in the *SERPINI1* gene, characterized by the intracellular deposition of polymers of neuroserpin (NS), which leads to proteotoxicity and cell death. Despite the different cell and animal models developed thus far, the exact mechanism of cell toxicity elicited by NS polymers remains unclear. Here, we report that human wild-type NS and the polymerogenic variant G392E NS form protein aggregates mainly localized within the endoplasmic reticulum (ER) when expressed in the yeast *S. cerevisiae*. The expression of NS in yeast delayed the exit from the lag phase, suggesting that NS inclusions cause cellular stress. The cells also showed a higher resistance following mild oxidative stress treatments when compared to control cells. Furthermore, the expression of NS in a pro-apoptotic mutant strain-induced cell death during aging. Overall, these data recapitulate phenotypes observed in mammalian cells, thereby validating *S. cerevisiae* as a model for FENIB.

## 1. Introduction

The intraneural accumulation of misfolded proteins is a common feature of several neurodegenerative pathologies such as Alzheimer’s, Parkinson’s and Huntington’s diseases, the incidence of which has been increasing with the increase in life expectancy. In these so-called conformational diseases [1], protein misconformers have the tendency to oligomerize and to form deposits that can induce cytotoxicity by alteration of the cellular homeostasis and biomolecular pathways [2,3]. Among the conformational diseases are the serpinopathies, referring to the human pathologies that are caused by mutations of serpins (serine protease inhibitors) [1]. Serpins are involved in many cellular processes, controlling many proteolytic cascades, including the mammalian coagulation pathways [4]. They are highly conserved in bacteria, archaea and eukarya, but rare in fungi and absent in yeast [5,6]. The serpin superfamily is divided into 16 clades, termed A to P. The majority of serpins inhibit serine proteases, but some of them also inhibit caspases and papain-like cysteine proteases [7]. Serpins are composed of the following different domains: 8–9 α helices (hA to hI), 3 β-sheets (A, B and C) and an exposed mobile reactive center loop (RCL) acting as a bait for the serine protease. The protease cleaves the RCL, triggering the insertion of the RCL into the β-sheet A. During this, the protease remains covalently bound to the serpin, as an acyl-enzyme intermediate, which comes along with a conformational change that alters the catalytic site and inactivates the protease.

Neuroserpin (NS, gene SERPINI1) is a neuronal serpin that inhibits the protease tissue plasminogen activator (tPA) [8,9]. Physiologically, NS is secreted from axons of both the central and peripheral nervous systems and is expressed in the late stages of neurogenesis during the process of synapse formation. In the adult brain, it is localized in areas where synaptic changes are associated with synaptic plasticity, learning and memory [10,11,12]. Point mutations affecting the structural flexibility of serpins also prevent their physiological role and often leading to serpin polymerization. Thus far, six different mutations, i.e., S49P, S52R, H338R, G392E, G392R and L47P [13,14,15,16], have been identified in NS and shown to be associated with the onset of FENIB (familial encephalopathy with neuroserpin inclusion bodies). The mutations prevent the normal trafficking and secretion of NS and promote the polymerization and intracellular retention of NS inside the endoplasmic reticulum (ER) of neurons, thereby causing cytotoxicity [17,18]. All NS mutants lead to the formation of inclusions called Collins bodies, and their amount directly correlates with the molecular instability of the mutated protein [13,19]. The Collin bodies show a globular shape with a size ranging from 5 to 50 μM in diameter, and they are mainly found in the neurons of the cortex, hippocampus and substantia nigra [13,14,15].

Although intracellular protein deposition is associated with neurodegeneration, the role of NS polymers in neurotoxicity needs additional investigation. NS polymers are highly ordered structures, and their formation is associated with the transient activation of the unfolded protein response (UPR) in mice models [20,21]. In cell models, however, NS polymers appear to activate the ER overload response (EOR), a stress signaling pathway that is independent of the UPR but that combines the accumulation of folded proteins in the ER with the calcium-dependent activation of NF-kB [22,23]. There is evidence that autophagy is involved in the degradation of all forms of NS, while ERAD (ER-associated degradation) selectively degrades non-polymerized mutant NS and other mutant serpin variants, such as α1-antitrypsin [24]. Since the protein quality control system and the mechanisms for protein clearance are gradually losing functionality during aging, the number of protein inclusions progressive increases, eventually leading to neuronal demise [25].

To date, several model systems have been developed to investigate FENIB, ranging from cell culture models [18,19,26,27,28,29], to transgenic flies [19], worms [21] and rodents [30,31,32]. Studies with these models confirmed a correlation between NS mutations, the retention in the ER and the accumulation of NS inclusions, suggesting that inclusion formation is the main factor driving neurodegeneration and disease symptoms. However, most studies did not evaluate the long-term effects related to cell demise. In fact, one study that used a HEK-293 cell model did not observe cell death when expressing the G392E NS mutant, which is known to trigger a severe phenotype in FENIB patients [33]. Additionally, another study that expressed the same mutant in neuronal progenitor cells (NPCs) obtained from an embryonic mouse cortex, did not report enhanced cell death in basal conditions but noticed that the G392E NS mutant promoted the expression of genes involved in oxidative stress, a process involved in several neurodegenerative disorders [26]. This incited us to develop a yeast model to further investigate the cellular phenotypes instigated by NS mutants.

The yeast *Saccharomyces cerevisiae* is, indeed, one of the validated biological model systems to study the pathobiology of human disease proteins and to elucidate the underlying molecular mechanisms and biochemical pathways [34,35,36,37,38]. Its main advantages are the reduced complexity compared to mammalian models, its manageability, low cost, the possibility of overcoming ethical problems, complete knowledge of its genome and the access to mutant libraries [39]. In this paper, we present data showing that the yeast model recapitulates important aspects related to the pathogenicity of neuroserpin, thereby offering a valuable tool to further decipher cell biological processes contributing to the aetiology of FENIB and to uncover new markers for diagnostics and targets for therapeutic strategies.

## 2. Materials and Methods

### 2.1. Yeast Strains and Culture Media

The *S. cerevisiae* wild-type and mutant strains used in this work are listed in Table 1. Yeast cells were grown under aerated and stirring conditions on an orbital shaker at 30 °C in YP medium (1% yeast extract, 2% bacto-peptone), or in minimal medium (6.7 g/L of yeast nitrogen base without amino acids), supplemented with different carbon sources (2% glucose; 2% galactose). Minimal media were supplemented with the required auxotrophies.

### 2.2. Plasmid Constructions

The cDNA of wild-type (WT) SERPINI1 and the G392E mutant were previously cloned in the Multiple Cloning Site (MCS) of the pTP6 expression vector [19,26,44]. These constructs served as a template to amplify both genes by PCR using a forward primer containing a BamHI site (CCCCCGGATCCATGGCTTTCCTTGGACTCTTCTC) and a reverse primer containing a HindIII site (CCCCCAAGCTTAAGTTCTTCGAAATCATGTCCAC). The amplification products were purified using the Gel/PCR DNA Fragments Extraction Kit (Geneaid New Taipei City, Taiwan) and inserted in the pUG35-URA vector to express the proteins as GFP-fusion under control of the methionine repressible MET17 promotor ((kindly provided by J. H. Hegemann, Heinrich-Heine-Universitat, Dusseldorf, Germany) or in the pESC-URA vector to express the native proteins under control of a galactose-inducible GAL1 promotor. The resulting yeast expression constructs were verified by sequencing.

### 2.3. Yeast Transformation

Yeast transformations were performed using the ONE-STEP protocol as previously reported [45]. Briefly, a colony grown on a YPD plate was resuspended in a reaction mixture containing 1 µg of plasmid DNA, 200 µL of PEG 60% (w/v), 60 µL of LiAc 1 M, 30 µL of DTT 1 M and 5 µL of salmon sperm DNA that served as a DNA carrier. This transformation mix was incubated 30 min at 45 °C, plated on selective glucose- and methionine-containing medium with the appropriate auxotrophies and growth at 30 °C for 3 to 4 days.

### 2.4. Growth Analysis

Cells were grown under non-inducing conditions at 30 °C for 24 h (h). The cells were collected, washed with sterile distilled water and re-inoculated in inducible medium to allow neuroserpin expression. As indicated, growth was monitored by measuring the OD600nm, either by taking samples from cultures grown in tubes or by using a Multiskan FC 96-well microplate spectrophotometer (Thermo Scientific, Merelbeke, Belgium). Four different transformants were taken for each construct and at least three independent experiments were performed. The doubling time was calculated as ln2 divided by the mean of the slopes (angular coefficient of the linear regression obtained from the plot of the log10 of the OD600 values).

### 2.5. Cell Viability Assay

In SD medium containing auxotrophic requirements as needed, 1 × 10^5^ cells/mL were inoculated and grown at 30 °C overnight. Cell viability was determined by the microcolony forming unit as described in [46].

### 2.6. H_2_O_2_ Test Assay

Yeast cells growing exponentially were exposed to the indicated H_2_O_2_ concentrations for 4 h at 30 °C. After treatment, cell viability was measured as described above.

### 2.7. Fluorescence Microscopy

Yeast transformant cells expressing fluorescently tagged proteins were analyzed using fluorescent microscopy with a Zeiss Axio Imager Z1 fluorescence microscope and the AxioVision 4.8 digital image processing system at a magnification of 63×. To visualize the endoplasmic reticulum, the cells were transformed with the FBp709 plasmid expressing the KAR2(1-135)-mCherry-HDEL (Kar2-mCherry) fusion protein from the TPI promoter as previously described [47]. The images of the cultures co-expressing NS and Kar2-mCherry were generated using a Leica DMi8 fluorescence microscope (Leica Microsystems Belgium BVBA, Diegem, Belgium) with specific filters. Pictures were deconvoluted with Huygens Essential software version 18.10 (Scientific Volume Imaging B.V., Hilversum, The Netherlands) and further processed with the standard ImageJ software. In addition, the ER was also visualized using the ER translocon subunit SEC63 fused to the GFP as a marker [48,49]. The quantifications of cells with neuroserpin foci were obtained by manual inspection of at least 200 cells per strain in two independent experiments.

### 2.8. Protein Extract

Whole cell protein extraction was performed using the TCA method, as previously described [50]. The mouse monoclonal anti-NS antibody was made in house as reported before [19] and immunodetected using enhanced chemiluminescence (ECL; SuperSignal‚ system, Pierce).

## 3. Results

### 3.1. Expression Levels and ER Localization of the NS Variants in Yeast Cells

**Figure 1:**
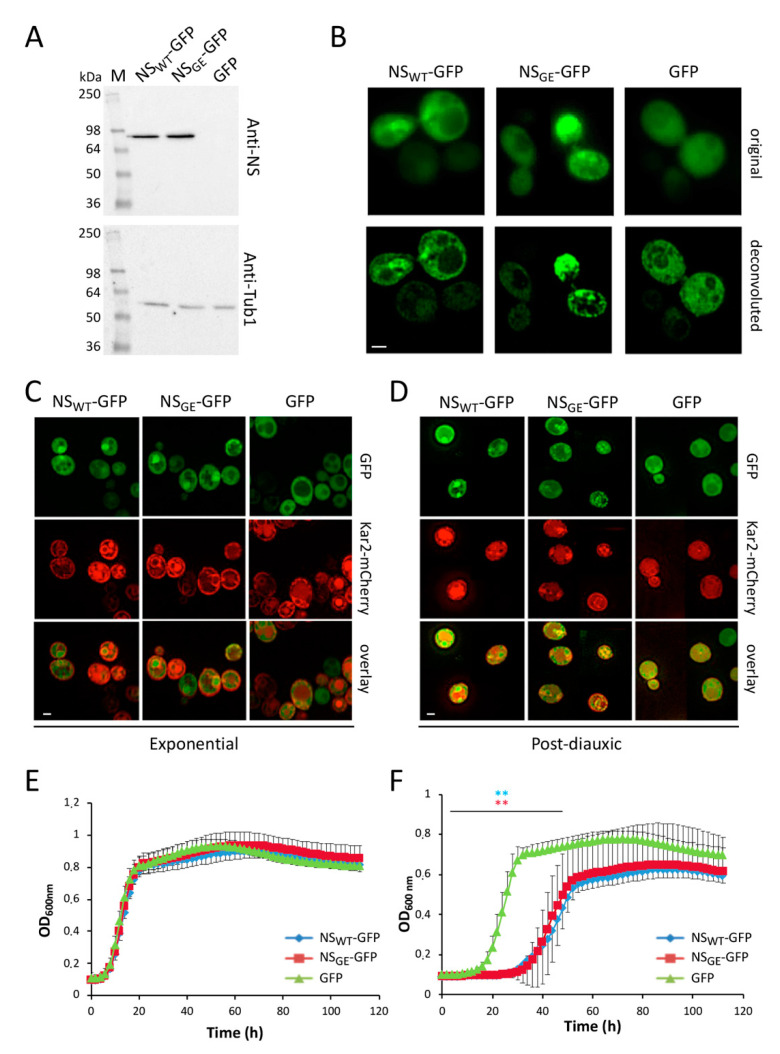
Human neuroserpin expressed in yeast cells forms ER-localized foci. (**A**) Protein extracts from BY4741 cells expressing NS_WT_-GFP, NS_GE_-GFP or GFP were separated on SDS-PAGE and probed with an anti-neuroserpin antibody (anti-NS; upper panel). The same filter, as a loading control, was probed with an anti-tubulin antibody (bottom panel). (**B**) Fluorescence microscopy pictures before and after deconvolution of BY4741 cells expressing NS_WT_-GFP, NS_GE_-GFP or GFP after 24 h of growth in inducing medium. (**C**,**D**) Representative fluorescence microscopy images of BY4742 cells co-expressing the ER marker Kar2-mCherry and GFP, NS_WT_-GFP or NS_GE_-GFP as indicated. Pictures were taken during the exponential (**C**) and post-diauxic (**D**) growth phases. Scale bar: 2 µm. (**E**,**F**) Growth curves of BY4742 yeast cells transformed with Kar2-mCherry and NS_WT_-GFP, NS_GE_-GFP or GFP in non-inducing medium (+methionine; (**E**)) and in inducing medium (-methionine; (**F**)). For each panel, the averages and standard deviations are shown based on three independent experiments. *p*-values: ** *p* < 0.01.

Human NS, both the wild-type and the mutated G392E genes, were expressed as GFP-fusion proteins, referred to as NS_WT_-GFP and NS_GE_-GFP, respectively, under the control of a methionine-repressible promotor in the yeast strains BY4741 and BY4742. A plasmid expressing the GFP gene was used as a control. Both the strains yielded similar results. The Western blot analysis showed a band of 82 kDa corresponding to the NS-GFP fusion proteins (Figure 1A), indicating that yeast cells efficiently express human NS. The fluorescence microscopy analysis revealed that while NS_WT_-GFP and NS_GE_-GFP were present in the cytoplasm, a significant fraction of the cells expressing NS_WT_-GFP and NS_GE_-GFP displayed foci and this in contrast to the control cells expressing native GFP (Figure 1B). To analyze this in more detail, NS_WT_-GFP or NS_GE_-GFP were co-expressed together with the Kar2-mCherry marker to visualize the peripheral and perinuclear ER in yeast because in mammalian cells, it is known that especially mutant NS is retained in the ER where it forms polymers [18,19,22,24,27,51]. This showed that during exponential growth foci were present in about one fifth (18.6%) of the cells expressing NS_WT_-GFP and in more than one fourth (26.7%) of the cells expressing NS_GE_-GFP (supplemental Figure S1). Interestingly, these foci were almost exclusively confined to the region of the nucleus and the perinuclear ER in the case of NS_WT_-GFP, while foci co-localized within, as well with, the peripheral ER in the case of NS_GE_-GFP. By the time the cells had traversed the diauxic shift to respire and to enter the stationary phase, the number of cells with foci had increased slightly and in about 22% of cells expressing NS_WT_-GFP and 31% of cells expressing NS_GE_-GFP, the foci co-localized with the perinuclear and peripheral ER (Figure 1C,D; Supplemental Figure S1). In the control cells, the native GFP was uniformly localized in the cytoplasm, not associated with a specific organelle and excluded from the vacuoles during the different growth phases.

### 3.2. Neuroserpin Expression Affects the Exit from Lag Phase and Cell Growth of Yeast

Growth analysis was performed to verify if the expression of NS_WT_-GFP or NS_GE_-GFP would trigger a cytotoxic effect. To this end, the cells were inoculated in non-inducing medium and allowed to grow overnight. Then, the cells were collected, washed and re-inoculated either in non-inducing (+methionine) or inducing (-methionine) medium at an OD600nm of 0.1. Cell growth was monitored for 72 h in a 96-well format. The cultures in non-inducing media showed a similar trend of growth during the time course, with similar duplication times (Figure 1E,F). When grown in inducing-medium, however, the cells expressing either NS_WT_-GFP or NS_GE_-GFP showed a delay in entering the logarithmic phase as compared to the cells expressing native GFP. Indeed, the cells expressing native GFP started to grow after 15 h, while those expressing NS_WT_-GFP or NS_GE_-GFP entered the exponential growth phase only after 30 h. Additionally, the duplication time was higher for the cells expressing NS_WT_-GFP or NS_GE_-GFP as compared to the GFP control. Finally, the cells expressing NS_WT_-GFP or NS_GE_-GFP reached the stationary phase at a lower cell density than those expressing GFP. The latter suggests that cells encounter problems when reprogramming from fermentation to respiration and entering the post-diauxic phase

### 3.3. Overexpression of Neuroserpin Confers Resistance to Oxidative Stress

**Figure 2:**
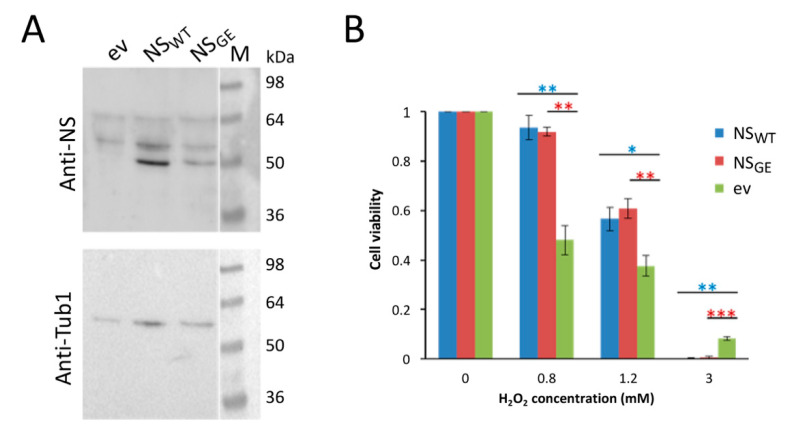
Neuroserpin expression protects cells from mild oxidative stress. (**A**) Western blot analysis of protein extracts of the BY4741 strain expressing native NS_WT_ or NS_GE_ under the control of the Gal1-10 promoter (pESC plasmid). Cells were grown on glucose until log phase and then shifted to galactose containing media for 24 h. NS detection was performed with an anti-neuroserpin antibody (anti-NS). The empty vector (ev) was used as a control. The same membrane was tested for the housekeeping protein tubulin (Tub1), as a loading control. (**B**) Cellular viability of BY4741 cells expressing NS_WT_ or NS_GE_. Viability is expressed as colony forming units (CFU) and was measured after exposure to H_2_O_2_ at the indicated concentrations for 4 h. The data are normalized to non-treated cells. The average of three independent experiments and standard deviation are presented. *p*-values: * *p* < 0.05; ** *p* < 0.01; *** *p* < 0.001.

The overexpression of NS in mouse embryonic progenitor cells differentiated to neurons induces a cellular stress response that protects them from further mild oxidative stress treatments [26]. Although the GFP constructs were very useful to localize NS within yeast cells, we expressed the wild-type and mutant neuroserpin as native proteins, without GFP-tag, under the control of a galactose-inducible promotor in the BY4741 strain in order to examine whether an effect on oxidative stress could also be demonstrated in yeast. Again, the expression of NS_WT_ and NS_GE_ was assessed using Western blot analysis. The results in Figure 2A show that, upon galactose shift for 24 h, both the NS variants were efficiently expressed and appeared as bands of about 50 kDa. Next, the cell viability was monitored before and after exposure to increasing concentrations of hydrogen peroxide (H_2_O_2_) to induce oxidative stress. This clearly revealed that the expression of both NS_WT_ and NS_GE_ conferred higher resistance to 0.8 and 1.2 mM H_2_O_2_ when compared to the control cells transformed with the empty vector (Figure 2B). However, higher concentrations of H_2_O_2_ (3 mM) were detrimental and almost completely killed all the cells expressing the NS variants, suggesting that neuroserpin only protects cells when challenged with a mild oxidative stress.

### 3.4. Overexpression of G392E Neuroserpin Triggers ER Aberrant Morphology

**Figure 3:**
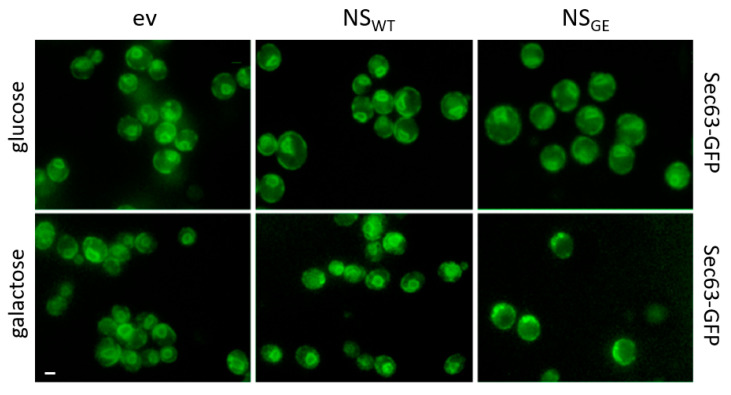
G392E neuroserpin expression induces aberrant ER morphology. Representative fluorescence microscopy images of BY4741 cells carrying the genomically tagged Sec63-GFP ER marker and co-expressing NS_WT_ or NS_GE_ from a galactose-inducible promotor. Cells were grown in glucose until log phase (SD) and then shifted to galactose containing media for 24 h. Bar: 2 μm.

During the Kar2-mCherry co-localization experiments, it was observed that a small number of cells displayed an abnormal ER network (Figure 1C,D). To further investigate a possible effect of NS on the ER structure and to confirm these data, we co-transformed the cells expressing native NS_WT_ or NS_GE_ with a plasmid containing the ER translocon subunit SEC63 fused to the GFP. This demonstrated that the defects in the ER morphology, visible as Sec63-GFP spots, were particularly associated with the expression of NS_GE_, as this was rarely seen upon expression of NS_WT_ and completely absent in the control cells (Figure 3).

### 3.5. Neuroserpin Expression Reduces Cell Viability in a Pro-Apoptotic Yeast Model

**Figure 4:**
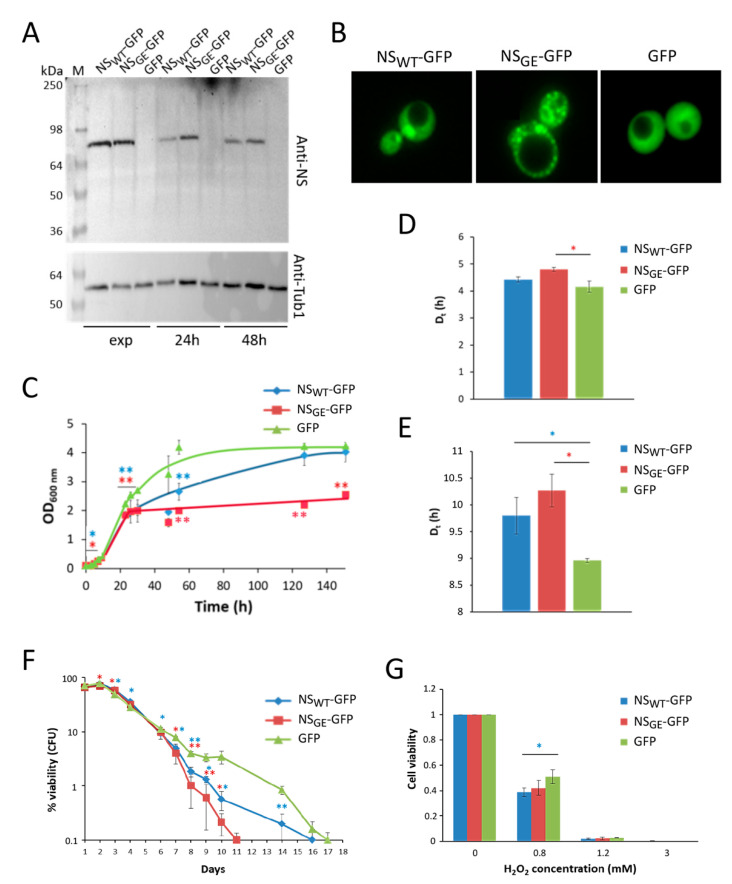
Neuroserpin shortens the lifespan of pro-apoptotic MCY4/Kllsm4Δ1 cells. (**A**) Western blot analysis of protein extracts obtained from MCY4/Kllsm4Δ1 cells expressing NS_WT_-GFP, NS_GE_-GFP or GFP sampled during the exponential phase (exp) and after 24 and 48 h of growth. Cells transformed with the empty vector served as control. For NS detection, membranes were probed with anti-neuroserpin antibody (anti-NS). The same membrane was tested for the housekeeping protein tubulin (Tub1), as a loading control. (**B**) Fluorescence microscopy of MCY4/Kllsm4Δ1 cells expressing NS_WT_-GFP, NS_GE_-GFP or GFP as indicated. (**C**) Growth curves of MCY4/Kllsm4Δ1 cells expressing NS_WT_-GFP, NS_GE_-GFP or GFP. (**D**,**E**) Duplication time (Dt) calculated from the cultures shown in panel (**C**) during the early (panel (**D**)) and late (panel (**E**)) exponential phase. (**F**) Viability of the MCY4/Kllsm4Δ1 mutant after induction of NS_WT_-GFP, NS_GE_-GFP or GFP expression during chronological aging. Viability is expressed as a percentage of colony-forming units (CFU) in a semilogaritmic scale. (**G**) Cell viability of MCY4/Kllsm4Δ1 mutant cells expressing NS_WT_-GFP, NS_GE_-GFP or GFP before and after exposure to the indicated H_2_O_2_ concentrations for 4 h. The data are normalized to non-treated cells. For each panel, the averages and standard deviations of three independent experiments are shown. *p*-values: * *p* < 0.05; ** *p* < 0.01.

To better characterize the phenotypes induced by the expression of wild-type and mutant NS, we made use of the yeast MCY4/Kllsm4Δ1 mutant strain, which shows a pro-apoptotic phenotype. In this strain, mRNA degradation is delayed, and it shows premature chronological aging accompanied by the presence of typical markers of regulated cell death: ROS accumulation, a large amount of oxidized RNA, and high sensitivity to oxidative stressing agents [40,41,52,53,54]. Furthermore, the Kllsm4Δ1 mutant cells also show an aberrant ER morphology [49]. Based on these phenotypes, we speculated that mRNA accumulation could accentuate the cellular toxicity associated to NS expression in yeast. To investigate this, we expressed the NS_WT_-GFP and NS_GE_-GFP fusion proteins in the MCY4/Kllsm4Δ1 strain, confirmed the expression during growth (Figure 4A), and analyzed the cultures using fluorescence microscopy. As shown in Figure 4B, especially upon expressing NS_GE_-GFP, foci were found in more than 35% of cells, which was again higher than upon expression of NS_WT_-GFP, where only 13% of cells displayed foci. Once more, the foci formed by NS_GE_-GFP and NS_WT_-GFP were differentially localized (Figure 4B). Hence, in general, these data were in agreement with those obtained with the BY4741 strain described above. Furthermore, similar observations were also made with the CML39-11A strain, which is the isogenic wild-type of the MCY4/Kllsm4Δ1 mutant (see Supplemental Figure S1). However, when performing the growth analysis to investigate the cytotoxicity triggered by NS, the data obtained with the Kllsm4Δ1 strain were slightly different, in that we did not observe a lag-phase extension upon NS_WT_-GFP and NS_GE_-GFP expression, although both NS variants still reduced the growth rate as compared to the expression of native GFP. In addition, the NS_GE_-GFP expressing cells arrested their growth once they had traversed the diauxic shift (Figure 4C–E). To better understand the effects of NS expression during aging, cell viability was also monitored during chronological aging. As shown, the MCY4/Kllsm4Δ1 mutant cells expressing NS_WT_-GFP and NS_GE_-GFP lost viability earlier compared to cells expressing native GFP, an effect that was more pronounced for NS_GE_-GFP than for NS_WT_-GFP (Figure 4F). Finally, viability was also tested after oxidative stress was triggered by treatment with different concentrations of hydrogen peroxide and in contrast to the BY4741 strain (Figure 2B), the expression of neuroserpin in the Kllsm4Δ1 strain made the cells more sensitive to H_2_O_2_ (Figure 4G), which is likely due to the fact that the MCY4/Kllsm4Δ1 strain is characterized by a higher intracellular ROS content [40,41,52,53,54]. For comparison, we also performed similar assays with the isogenic wild-type strain, CML39-11A. Similar to that observed in the BY4741 strain, the expression of NS_WT_-GFP or NS_GE_-GFP triggered an extension of the lag phase and an increase in the duplication time as compared to the expression of native GFP, though all the cultures reached a similar density in the stationary phase (Supplemental Figure S2A). In addition, the CML39-11A cells expressing either NS_WT_-GFP or NS_GE_-GFP were characterized by a significantly shortened chronological lifespan (Supplemental Figure S2B), while they were only slightly more resistant to H_2_O_2_ (Supplemental Figure S2C).

## 4. Discussion

Over the past decade, the budding yeast *S. cerevisiae* has been extensively used as a powerful model for accelerating and facilitating the identification of genetic players involved in the development of neurodegenerative disorders characterized by protein misfolding and protein aggregation. Yeast cells are less complex than human neurons but, nevertheless, the basic molecular pathways are well-conserved. Moreover, the availability of mutant yeast libraries, the ease of handling and its fast growth make *S. cerevisiae* an excellent tool to gain insight into the cellular mechanisms underlying human disorders, including neurodegenerative diseases [34,35,36,37,38]. One type of neurodegenerative disease is FENIB, of which the etiology is associated to mutations in the SERPINI1 gene causing the encoded NS proteins to have a higher tendency to polymerize and to be retained in the ER. The G392E mutation, especially, promotes one of the most severe FENIB phenotypes [13,19]. In this paper, we present the first humanized yeast FENIB model that, in support of existing mammalian cell and animal models, can enable the mechanisms involved in NS polymer toxicity to be deciphered.

We investigated the effects of the wild-type (NS_WT_) and the G392E (NS_GE_) NS variants in different *S. cerevisiae* strains using two different expression systems, a methionine-dependent expression as GFP-fusion transcription and a galactose-dependent expression of the untagged native proteins. Although NS_WT_ and NS_GE_ were present in the cytoplasm and nucleus, we were able to observe that both neuroserpin variants formed ER-localized foci in the different yeast strains. This is in agreement with previous observations in neurons of patients affected by FENIB [13,14,19] and in accordance with other model systems, including different cell lines [13,14,16,18,19,22,26,27,28] as well as transgenic models, such as worms [21], flies [19] and mice [30,32]. The fact that also NS_WT_ formed intracellular foci in our yeast system could be due to the high levels of expression and/or to an inefficient secretion of this protein in yeast. Indeed, we were not able to detect the presence of NS_WT_ in the culture medium of the yeast cells (results not shown). It has been reported that wild-type alpha-1 antitrypsin, also responsible for a pathology caused by polymer formation [55], undergoes a low degree of polymerization when compared to disease-causing variants and if expressed at high levels in mammalian cells [56]. Accordingly, in yeast cells the number of cells displaying visible foci formed by NS_WT_ was lower as compared to cells expressing the NS_GE_ mutant, suggesting that the latter is more prone to also polymerize in yeast. A similar conclusion could also be drawn from the observation that, already during exponential growth, NS_GE_ started to form foci that co-localized with the peripheral ER and thereby more foci were present per cell during this growth phase as compared to NS_WT_, whose foci were then still confined to the perinuclear ER. Whether this means that NS_GE_ has a higher seeding capacity than NS_WT_, remains to be investigated. Additionally, the nature of the foci needs to be studied in more detail, since it may well be that the type of aggregation and the solubility of the formed aggregates may differ between NS_GE_ and NS_WT_. Nonetheless, our data clearly showed that the expression of both NS variants affected the growth and viability of the yeast cells, indicating that the formation of NS inclusions triggers cytotoxicity, presumably by challenging the mechanisms involved in protein quality control and the clearance of misfolded conformers. Especially in the case of NS_GE_, this was accompanied by an aberrant ER morphology that appeared fragmented and clustered, reminiscent of the vesiculated ER observed in mammalian cells overexpressing G392E NS [16,19,26]. This phenotype is probably associated to the ER stress caused by the protein burden that the ER has to face when NS accumulates within the organelle. It is well established that the ER is a site for the generation of reactive oxygen species (ROS) besides mitochondria. In the first instance, this is due to an upregulation of the oxidative protein folding machinery that is controlled by the UPR and, in case of sustained ER stress, the enhanced ROS production can become detrimental for the cells [57]. It has been argued that NS polymers do not trigger the UPR, but instead lead to an ER overload and activation of the EOR pathway that is associated with the detection of Ca^2+^ perturbations and that also involves ROS signaling [22,23]. Whether a true equivalent of the EOR pathway is present in yeast has not been reported, but it is known that during ER stress Ca2+ is released from the ER to enter mitochondria where it is suggested to initiate the activation of a mitochondrial adaptive response that allows coping with moderate levels of ER-stress and that is linked to an increased O_2_ consumption, increased mitochondrial membrane potential and an enhanced respiratory capacity [58,59,60,61].

In mouse neural progenitor cells, it has been reported that the G392E NS mutant induces an antioxidant response that increases cell resistance to oxidative stress [26]. We observed a similar protective effect, especially in NS-expressing BY4741 cells treated with low doses of H_2_O_2_, albeit in this case the effect was observed with both NS_WT_ and NS_GE_. The phenomenon is known as mitochondrial hormesis [62] and it is linked to the mitochondrial adaptive response described above. Importantly, the hormesis response is only effective when cells encounter a manageable dose of stress. Indeed, when the BY4741 cells were treated with higher doses of H_2_O_2_ or when a low dose is given to the Kllsm4Δ1 mutant strain that has already enhanced levels of ROS, NS expression becomes detrimental, probably because the additional ROS that is formed by NS-induced ER-stress leads to an ROS overproduction that can no longer be handled by the oxidative stress defense system. In line with this is the observation that neural progenitor cells expressing G392E NS display a higher susceptibility to apoptosis upon inhibition of the antioxidant defenses [26].

The hormesis effect also likely explains the difference in growth profiles between the different strains used in this study, where the BY4741 and CML39-11A strains display a prolonged lag phase upon NS expression because they still need to adapt and activate the responses associated to enhanced ER stress, while in the MCY4/Kllsm4Δ1 strain, these adaptive responses are already activated, allowing the NS-mediated ER stress to be immediately handled and growth to start.

As for other neuropathies, aging plays a crucial role in the onset and progress of FENIB disease: the accumulation of NS polymers comes with an increasing burden for the aging organism as seen for worms [21], flies [19] and mice [30,32]. In fact, in the D. melanogaster model for FENIB, the flies expressing wild-type and mutant G392E NS were similar at eclosion and had similar lifespans, but those expressing the G392E mutant presented a progressive reduction in motor function in direct correlation with the level of NS polymers accumulated in the brain [19]. Several studies indicated that the chronological aging of non-dividing stationary yeast cells mimics the aging process of post-mitotic mammalian cells such as neurons [63,64]. While both NS_WT_ and NS_GE_ had an impact on yeast longevity, we noted that especially the expression of mutant NS_GE_ in the MCY4/Kllsm4Δ1 strain, led to a significantly shortened lifespan. Interestingly, in this strain, NS_GE_ formed markedly more foci than NS_WT_ and the mutant arrested cell growth when entering the diauxic shift. This suggests that a correlation between the accumulation of NS polymers, cytotoxicity and enhanced cell demise during aging is also seen in yeast. However, it appears that the preadaptation of the cells to enhanced levels of ROS is also an important factor, since, in contrast to the MCY4/Kllsm4Δ1 strain, both NS_WT_ and NS_GE_ triggered a similar shortening of the chronological lifespan in the CML39-11A wild-type strain.

In conclusion, our data validate the use of yeast as a model for future research on serpinopathies such as FENIB, where particularly yeast genetic screens will help to further dissect the processes affected by NS polymerization and to identify potential markers for diagnosis or targets for therapeutic intervention.

## Figures and Tables

**Table 1:** Strains used in this study.

Strain	Genotype	Reference
CML39-11A	*Mat a*, *ade1-101*, *his3-Δ1*, *leu2*, *ura3*, *trp1-289*	[40]
MCY4/Kllsm4Δ1	*Mat α*, *ade1-101*, *his3-Δ1*, *trp1-289*, *ura3*, *LEU2-GAL1-SDB23 pRS313/Kllsm4Δ1*	[41]
BY4741	*Mat a*, *his3-Δ1*, *leu2-Δ0*, *met15-Δ0*, *ura3-Δ0*	[42]
BY4742	*Mat α his3-Δ1 leu2-Δ0 lys2-Δ0*, *ura3-Δ0*	[42]
BY4741 Sec63-GFP	*Mat a*, *his3-Δ1*, *leu2-Δ0*, *met15-Δ0*, *ura3-Δ0 Sec63-GFP*	[43]

## Supplementary Materials

The following are available online at https://www.mdpi.com/article/10.3390/microorganisms9071498/s1, Figure S1: Percentage of cells displaying foci of NS_WT_-GFP or NS_GE_-GFP Figure S2: Expression of NS_WT_-GFP or NS_GE_-GFP in CML39-11A strain.
