# Supplementary material for: Neuroserpin Inclusion Bodies in a FENIB Yeast Model
Source: Microorganisms. 2021 Jul 13;9(7):1498. doi: 10.3390/microorganisms9071498 (PMC8305157; doi:10.3390/microorganisms9071498)

microorganisms

Supplementary figures

Figure S1: Percentage of cells displaying foci of NS_{WT}-GFP or NS_{GE}-GFP when expressed in the BY4741 (A) and CML39-11A (B) wild type strains and the MCY4/Kllsm4Δ1 mutant strain (C). The quantifications of cells with neuroserpin foci were obtained by manual inspection of at least 200 cells per strain in two independent experiments.

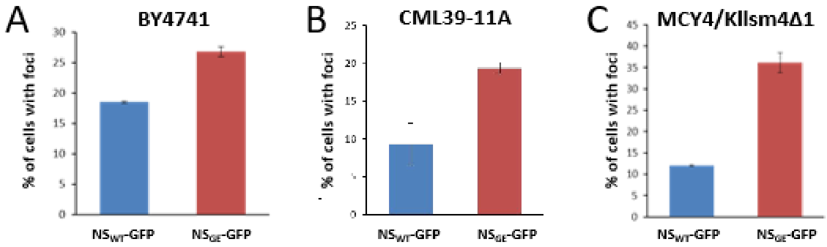

Figure S2: Growth (A), chronological lifespan (B) and H_2O_2 resistance (C) of CML39-11A cells expressing either NS_{WT}-GFP, NS_{GE}-GFP or native GFP as indicated. For H_2O_2 resistance, the viability was monitored by counting the colony forming units (CFU) and was measured after exposure to H_2O_2 at the indicated concentrations for 4 h. The average of three independent experiments and standard deviation are presented. P-values: *p <0,05.

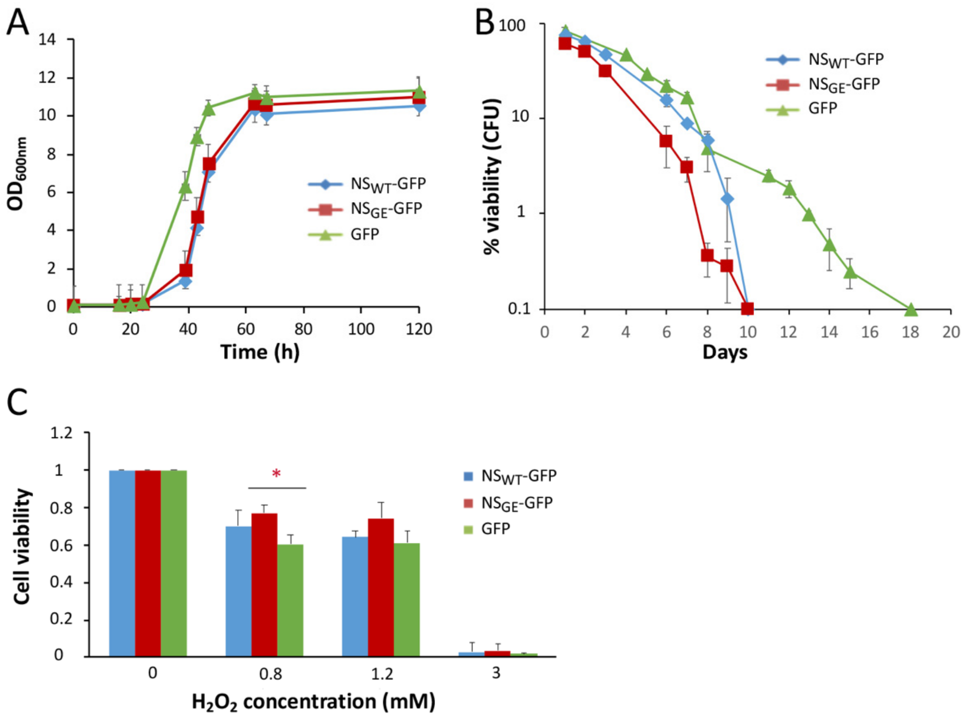

Supplement: Supplementary file 1 [file microorganisms-09-01498-s001.zip › microorganisms-1275919-supplementary.pdf]
